# Correlation of student performance on clerkship with quality of medical chart documentation in a simulation setting

**DOI:** 10.1371/journal.pone.0248569

**Published:** 2021-03-15

**Authors:** Nobuyasu Komasawa, Fumio Terasaki, Takashi Nakano, Ryo Kawata

**Affiliations:** Medical Education Center, Osaka Medical College, Osaka, Japan; Lundquist Institute at Harbor-UCLA Medical Center, UNITED STATES

## Abstract

**Background:**

Medical chart documentation is an essential skill acquired in a clinical clerkship (CC). However, the utility of medical chart writing simulations as a component of the objective structured clinical examination (OSCE) has not been sufficiently evaluated. In this study, medical chart documentation in several clinical simulation settings was performed as part of the OSCE, and its correlation with CC performance was evaluated.

**Methods:**

We created a clinical situation video and images involving the acquisition of informed consent, cardiopulmonary resuscitation, and diagnostic imaging in the emergency department, and assessed medical chart documentation performance by medical students as part of the OSCE. Evaluations were conducted utilizing original checklist (0–10 point). We also analyzed the correlation between medical chart documentation OSCE scores and CC performance of 120 medical students who performed their CC in 2019 as 5^th^ year students and took the Post-CC OSCE in 2020 as 6^th^ year students.

**Results:**

Of the OSCE components, scores for the acquisition of informed consent and resuscitation showed significant correlations with CC performance (P<0.001 for each). In contrast, scores for diagnostic imaging showed a slightly positive, but non-significant, correlation with CC performance (P = 0.107). Overall scores for OSCE showed a significant correlation with CC performance (P<0.001).

**Conclusion:**

We conducted a correlation analysis of CC performance and the quality of medical chart documentation in a simulation setting. Our results suggest that medical chart documentation can be one possible alternative component in the OSCE.

## Introduction

A strong medical education values, above all else, the ability for undergraduate through postgraduate students to hone their skills so that they become trusted healthcare professionals [1, 2]. To this end, medical educators must establish an effective clinical training curriculum for undergraduate students in medical school that allows them to move seamlessly into their basic skill training as postgraduates [3].

In Japan, clinical training takes the form of a clinical clerkship (CC) [4], which differs from conventional observation-based clinical training. A CC student is a member of the medical team and participates in actual medical practice and care with supervising doctors [5]. Because students are allowed to perform a certain range of medical procedures under the guidance and monitoring of a teaching doctor [6, 7], they are able to acquire practical clinical skills. Students are required to acquire basic physical examination and precise medical chart documentation skills, as well as the ability to present at conferences. Clinical training in diagnoses and treatment for CC students follows a curriculum that is determined by each hospital department [8].

Physical examinations and medical chart documentation are among the most important skills learned in a CC [9]. Medical chart documentation is essential not only from legal and insurance (e.g., providing evidence for insurance) perspectives, but also for cultivating clinical abilities such as making a differential diagnosis [9].

In 2005, with the intent to ensure basic clinical competency in medical students, the Common Achievement Test Organization (CATO) was established as a third party and introduced the objective structured clinical examination (OSCE) and computer-based testing (CBT) to evaluate basic medical knowledge [10]. Both the OSCE and CBT are required for the Association of Japanese Medical Colleges to recognize a medical student as a ‘student doctor.’ The Pre-CC OSCE (conducted prior to the CC) evaluates basic clinical competency. In 2020, the Post-CC OSCE, which evaluates clinical competency after CC, was introduced as well. A strong performance on this examination is required to graduate from medical schools in Japan.

While the above tests are used to evaluate clinical competencies such as medical interviews and physical assessments, only a few tests offer a comprehensive evaluation of a student’s medical chart documentation skills after CC completion [11, 12]. Furthermore, no study to date has examined correlations between medical chart documentation performance and CC performance. Therefore, we created a medical chart documentation OSCE and assessed its relationship with CC performance in the context of medical education in Japan. In this study, we developed three simulation-based medical chart documentation OSCE components for Post-CC students and analyzed correlations between their performance on the medical chart documentation OSCE and CC.

## Methods

### Ethical considerations

This study was approved by the Research Ethics Committee of Osaka Medical College (No.2806-1). Verbal informed consent was obtained from students by medical teacher and clerks also witnessed the process. All students were informed about the nature and purpose of the study and anonymity was guaranteed. Students were also informed that they had the opportunity to withdraw from the study if they notified the investigator within a week after taking the OSCE. We also emphasized that withdrawing from the study would not influence their academic outcomes in any way. There were no minors in the study population, since all 6^th^ year medical students in Japan are aged >23 years.

### Settings

As is the case for most medical schools in Japan, Osaka Medical College requires its students to take the Pre-CC OSCE and CBT in their 4^th^ year, before they enter into CC in their 5^th^ and 6^th^ years. The Pre-CC OSCE included the following seven basic clinical components: medial interview, chest examination, abdominal examination, head and neck examination, neurological examination, basic clinical technique, and emergency response.

**Fig 1:**
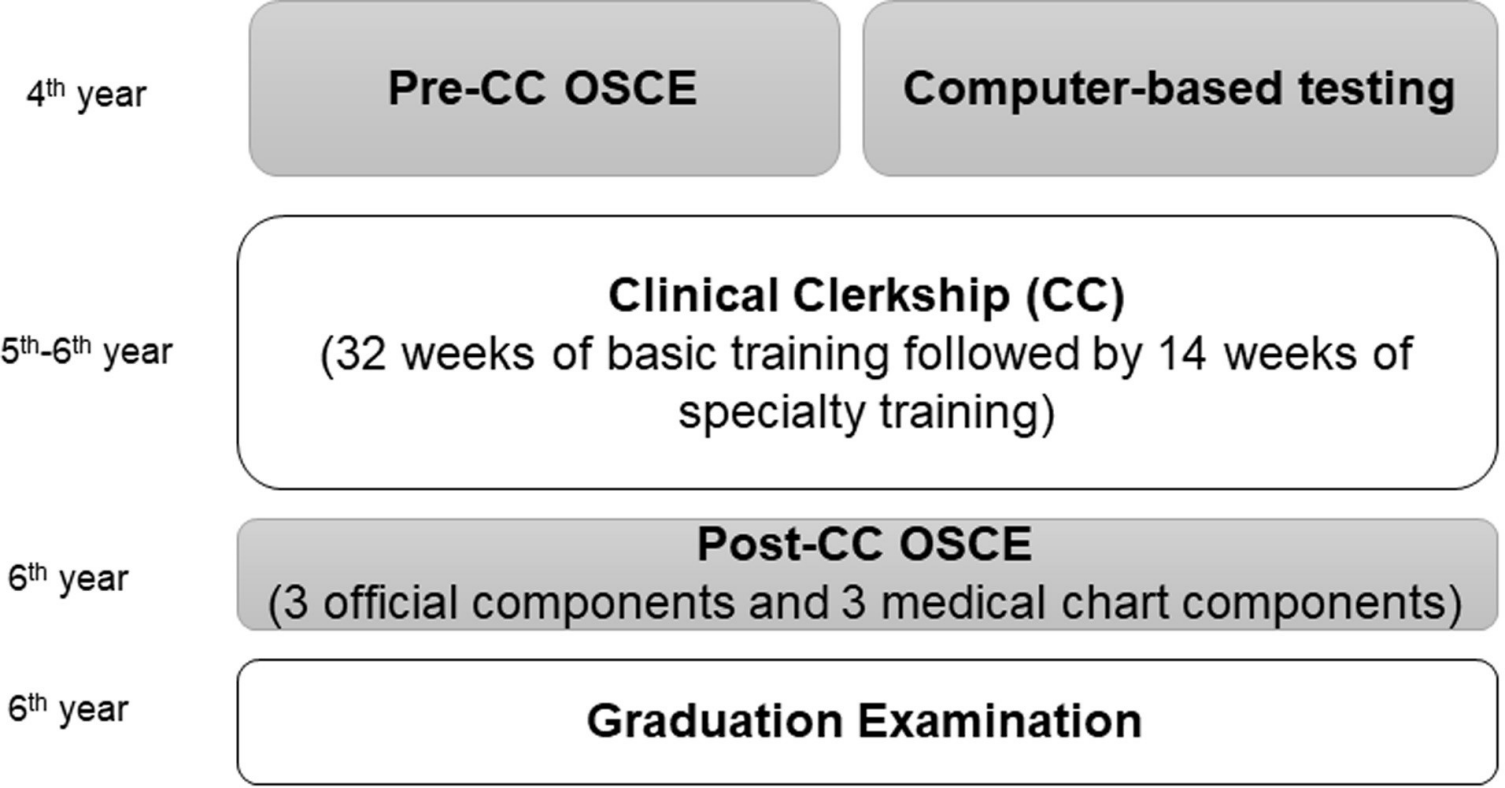
Timeline of medical student curriculum as related to the Objective Structured Clinical Examination (OSCE) and Clinical Clerkship (CC). In 2020, we assessed only the official components for the post-CC OSCE due to the COVID-19 pandemic.

In 2020, the Post-CC OSCE was introduced as a way to evaluate the clinical skills cultivated in CC (Fig 1). The Post-CC OSCE comprises three official mandatory components provided by the CATO in Japan and three original components created by each medical college. Each medical school must perform the three official mandatory components and at least three original components. Mandatory components include a medical interview, physical examination, and presentation. We considered medical chart documentation to be an essential skill for medical students and developed three medical chart simulation components as original components to include in the Post-CC OSCE.

### Study population

We recruited 120 students of Osaka Medical College who were in their 5^th^ year in 2019 and 6^th^ year in 2020. Repeat-year students in the 2019 curriculum year were excluded because the evaluators of student performance are generally not the same every year.

### Study measures

#### Post-CC OSCE original components

All 120 medical students were tested on the 3 original components of informed consent, resuscitation, and diagnostic imaging, as discussed below.

The first original component involved documenting the content of a five-minute video in which medical doctors and nurses go through the process of obtaining informed consent from various patients. We created four scenarios: surgical explanation, palliative care introduction, general anesthesia, and emergency response. One of these was randomly selected for the Post-CC OSCE [13]. After watching the video twice, students were given 10 minutes to document whatever information they felt was relevant by free description on a paper note using the standard format they were taught in CC training. With regard to the content of informed consent, they were instructed to document essential information such as patient condition, treatments, risks, and questions from the patient.

The second original component involved documenting the content of a five-minute video on a case requiring resuscitation. In the video, medical doctors used manikins to perform advanced life support for an adult patient and basic life support for a pediatric patient [14]. One of three available resuscitation scenarios was randomly selected and used for the Post-CC OSCE. After watching the video twice, students were given 10 minutes to document relevant information in the manner described above for the first original component. They were also instructed to document essential information about each situation or each treatment during resuscitation.

The third original component involved diagnostic imaging in the emergency department. We created a five-minute clinical presentation video in which age, sex, height and weight, past and current medical history, symptoms, and physical assessment were first presented and followed by several images (scenarios) of conditions requiring an emergency response (brain hemorrhage, tension pneumothorax, and cardiac tamponade) [15]. One of these scenarios was selected randomly for the Post-CC OSCE. Here too, students watched the video and images twice and were given 10 minutes to document essential information regarding the patient, differential diagnosis, and treatment.

#### Medical chart evaluation utilizing

Student performance was evaluated by double-blinded examiners using original checklist (0–10 points) (Table 1). These checklists were developed by 3 clinical educators (authors NK, FT, and RK) with practical experience in evaluating the quality of student documentation. In developing the checklists, essential elements of documentation were determined by consensus and also by referring to previous studies [16, 17]. Evaluations were performed by one teacher (author NK) who was a Certified Healthcare Simulation Educator and a Japan Society for Medical Education Certified Medical Education Specialist. Checklist evaluations were performed by one teacher to ensure consistency.

**Table 1:** Medical chart documentation checklist in each component.

	Component 1 Informed consent	Component 2 Resuscitation	Component 3 Diagnostic imaging
**1**	Date, place	Date, place	Date, place
**2**	Patient or family name	Patient name	Patient name
**3**	Explainer, Attender	Present history	Present history
**4**	Patient condition	Electric treatment	Disease name
**5**	Content of medical procedure	Drug treatment	Image findings
**6**	Alternative description	Type of cardiac arrest	Left or Right
**7**	Complications	Differential diagnosis	Initial treatment
**8**	Understanding by patient or family	Communication with medical staff members	Differential diagnosis
**9**	Question and answer	Time course	Time course
**10**	Organized	Organized	Organized

#### CC content and evaluation

During their 5^th^ year, medical students enter into a basic CC, in which they participate in the CC for all clinical departments of the hospital over the course of 32 weeks. Each CC spans roughly 1–2 weeks in duration. Once the basic CC is completed, students must then select a discipline they wish to study for 14 weeks from the end of their 5^th^ year to early in their 6^th^ year (Fig 1).

During each CC, supervising (teaching) doctors of each department evaluate the clinical skills of students using an evaluation sheet based on the mini-clinical evaluation exercise (CEX) and direct observation of procedural skills (DOPS) [18, 19]. This assessment comprises a 5-point evaluation sheet with 16 items (80%), subjective evaluation by the organizer of each department (10%), and a written report (10%).

Scores for each CC are collected by the medical education center and used to calculate an average score. In this study, we focused on the basic CC (32 weeks) score, since all medical students follow the same curriculum during their basic CC [20].

### Statistical analysis

Statistical analysis was performed using JMP^®^ 11 (SAS Institute Inc., Cary, NC, USA). Results were compared using Pearson’s correlation test. Data are presented as mean ± SD. P < 0.05 was considered statistically significant.

### Patient and public involvement

Neither patients nor the public were involved in the design, execution, reporting, or dissemination of this study.

## Results

The Post-CC OSCE was performed on July 7, 2020. Due to the COVID-19 pandemic, we were unable to perform the mandatory components of CATO; however, we were able to perform our three original medical chart documentation components. None of the medical students withdrew from the study.

For the 2020 Post-CC OSCE, we selected informed consent for general anesthesia as Component 1, adult advanced life support as Component 2, and diagnostic imaging for cardiac tamponade as Component 3. We analyzed data from 120 medical students who took the Post-CC OSCE in 2020 and who had undergone the basic CC in 2019. Medical students scored between 70–90% on each component (Table 2).

**Table 2:** Medical student scores for Clinical Clerkship (CC) and Post-CC Objective Structured Clinical Examination (OSCE).

	Component 1 Informed consent (0–10)	Component 2 Resuscitation (0–10)	Component 3 Diagnostic imaging (0–10)	Total Score (0–30)	Clinical Clerkship
Average	7.2	7.2	7.4	22.0	78.3
SD	1.4	1.2	1.1	2.2	2.5

Correlations between clinical competency scores and medical chart documentation scores of the OSCE are shown in Table 3. With regard to OSCE components, the acquisition of informed consent and resuscitation showed significant correlations with a student’s CC performance (P<0.001 for each), and diagnostic imaging showed a slightly positive, but non-significant, correlation (P = 0.107). When all OSCE scores were combined, the correlation was statistically significant (P<0.001).

**Table 3:** Correlations between medical chart documentation scores from the Objective Structured Clinical Examination (OSCE) and Clinical Clerkship (CC).

	Component 1 Informed consent	Component 2 Resuscitation	Component 3 Diagnostic imaging	Total Score
R	0.813	0.729	0.148	0.749
Co-efficient	0.661	0.532	0.013	0.561
P	<0.001	<0.001	0.107	<0.001

*P<0.05.

## Discussion

One main objective of most medical schools worldwide is providing students with an education that prepares them to transition seamlessly from the stage of knowledge acquisition to performing practical skills in clinical settings. Accordingly, medical education programs aim to produce competent graduates who can record patient history through medical interviews, conduct a comprehensive physical examination, and perform medical chart documentation. Of these competencies, medical chart documentation skills are considered to be very important [20, 21].

Patient interviews and medical chart documentation are essential skills, and accurate documentation and evaluation allow medical students to collect the information required for appropriate diagnosis and treatment during their CC [22, 23]. In the clinical setting, it is not rare to miss abnormal physical findings or to perform a critical evaluation incorrectly. Incorrect medical chart documentation can lead to diagnostic errors, which can result in an adverse outcome for the patient. If medical students are to acquire clinical competency and become trustworthy healthcare professionals, then they must be taught and expected to perform both technical and non-technical skills at a very high level [24].

Medical students must practice medical chart documentation before moving on to post-graduate training. In their CC rotations, medical students are expected to be active participants in the healthcare team, interviewing patients, documenting complaints and findings, and serving as patient advocates by communicating patient issues to the team. In these ways, students facilitate information sharing among healthcare team members [25]. Students also acquire competency by practicing and receiving feedback on their clinical skills, including the ability to fill out medical charts and reason through diagnostic and therapeutic plans [26]. The medical record is a critical resource for promoting clinical competency among medical students because it provides access to patient information and educational resources. It also serves as an important venue for assessing medical student competencies. In fact, medical record review is one of the assessment tools described in the Accreditation Council for Graduate Medical Education toolbox for guiding competency assessment in the six core domains [27, 28].

In our study, overall OSCE scores showed a strong and significant correlation with medical student performance with regard to informed consent and resuscitation components, but showed no significant correlation with performance on the emergency diagnostic imaging component. This may be attributed to the fact that during their CC, medical students participate regularly in situations involving acquisition of informed consent, and advanced life support training is becoming common in simulation training during the CC. In contrast, medical students are less likely to have direct clinical experience with emergency diagnostic imaging. Accordingly, the inclusion of emergency diagnostic imaging using a problem-based learning simulation in the curriculum may be warranted. While our medical chart documentation OSCE does not require human-to-human contact, it may be effective for summative evaluation during pandemic situations such as COVID-19.

This study has several limitations worth noting. First, we performed a summative evaluation of OSCE and CC performance in a single number though medical students rotate through so many subject areas, are assessed on so many skills. It would be more relevant to evaluate correlations with validated measures of documentation quality or future (and not past) performance. Furthermore, we evaluated overall scores for the CC rather than those for individual components. Assessing correlations among various evaluation aspects during CC may further clarify relationships with performance on the medical chart documentation OSCE. Second, we developed the original checklists based on previous studies and one teacher performed all evaluations. It will be important to validate the checklists to ensure their generalizability and to have multiple evaluators perform the evaluations. Third, all documentation by students was made on paper because we believe paper documentation to be a fundamental skill. However, given the spread of electric medical records, it will be important to include electric medical chart documentation scenarios in the future. Finally, as the data came from a single institution, our findings may not be generalizable to other medical schools. However, we believe that our results can be applied to most other Japanese medical schools as they all follow the main core curriculum provided by the Ministry of education.

In this study, we performed a correlation analysis of student performance on CC with the quality of medical chart documentation in a simulation setting. Our results suggest that medical chart documentation can be one possible alternative component in the OSCE.

## Supporting information

S1 Data (XLSX)
